# Development and validation of a nomogram for preoperative prediction of lymph node metastasis in early gastric cancer

**DOI:** 10.1186/s12957-019-1778-2

**Published:** 2020-01-02

**Authors:** Xiao-Yi Yin, Tao Pang, Yu Liu, Hang-Tian Cui, Tian-Hang Luo, Zheng-Mao Lu, Xu-Chao Xue, Guo-En Fang

**Affiliations:** 1 Department of General Surgery, Changhai Hospital, The Second Military Medical University, 168 Changhai Road, Shanghai, 200433 China; 2 Department of Gastroenterology, Changhai Hospital, The Second Military Medical University, Shanghai, 200433 China

**Keywords:** Early gastric cancer, Lymph node metastasis, Nomogram, Preoperative prediction

## Abstract

**Background:**

The status of lymph nodes in early gastric cancer is critical to make further clinical treatment decision, but the prediction of lymph node metastasis remains difficult before operation. This study aimed to develop a nomogram that contained preoperative factors to predict lymph node metastasis in early gastric cancer patients.

**Methods:**

This study analyzed the clinicopathologic features of 823 early gastric cancer patients who underwent gastrectomy retrospectively, among which 596 patients were recruited in the training cohort and 227 patients in the independent validation cohort. Significant risk factors in univariate analysis were further identified to be independent variables in multivariable logistic regression analysis, which were then incorporated in and presented with a nomogram. And internal and external validation curves were plotted to evaluate the discrimination of the nomogram.

**Results:**

Totally, six independent predictors, including the tumor size, macroscopic features, histology differentiation, P53, carbohydrate antigen 19-9, and computed tomography-reported lymph node status, were enrolled in the nomogram. Both the internal validation in the training cohort and the external validation in the validation cohort showed the nomogram had good discriminations, with a C-index of 0.82 (95%CI, 0.78 to 0.86) and 0.77 (95%CI, 0.60 to 0.94) respectively.

**Conclusions:**

Our study developed a new nomogram which contained the most common and significant preoperative risk factors for lymph node metastasis in patients with early gastric cancer. The nomogram can identify early gastric cancer patients with the high probability of lymph node metastasis and help clinicians make more appropriate decisions in clinical practice.

## Background

Gastric cancer (GC) ranks the fifth in the most common cancer in the world, which is the third most common cause of death related to cancer worldwide [1]. Because of the absence of typical symptoms, most GC patients are diagnosed at advanced stage which leads to a poor prognosis. It was reported that the 5-year survival rate was lower than 25% [2]. However, with the progress of public health program and the popularization of gastroscopy in primary hospitals, more patients with early gastric cancer (EGC) are diagnosed and their 5-year survival rate reaches over 90% in Japan and Korea [3].

With the development of endoscopic therapy, most EGC can be effectively treated by minimum invasive endoscopic treatments, such as endoscopic mucosal resection (EMR) and endoscopic submucosal dissection (ESD), which can better preserve gastric function and reduce complications and cost compared with surgical operation [4–6]. However, a meta-analysis showed that the tumor recurrence after ESD is higher than surgical resection [7], whose reasons are related to metachronous new primary tumors, non-curative ESD, synchronous multiple primary tumors [8], and occult lymph node metastasis (LNM) before the operation [9]. Therefore, endoscopic treatment should be suggested under the circumstance that the possibility of LNM is exceedingly low, and both the lesion size and site of the EGC are suitable for whole resection [10]. Besides, LNM in EGC patients is an important indication for the extent of lymphadenectomy. Patients with cT1N0 GC should be recommended to undergo a D1 or a D1+ lymphadenectomy, and a D2 lymphadenectomy is suggested for patients with cT1N+ tumors, according to the Japanese Gastric Cancer Treatment Guideline [11].

Hence, accurate identification of LNM in patients with EGC is critical to patients’ prognosis and treatment decision [12]. But the probability of LNM is still evaluated by the general guidelines and surgeon’s experience without quantified standards in clinical practice nowadays. Although several studies have tried to explore the risk factors of LNM in EGC, most items involved, such as lymphovascular invasion and depth of tumor invasion, were unavailable preoperatively [13, 14]. The comprehensive analysis of gastroscopic findings, tumor markers, and radiology images, rather than individual analyses, is the most promising way to improve clinical management [15]. To our knowledge, there are no studies containing only preoperative factors to predict the probability of LNM in EGC.

Therefore, the present study aimed to analyze risk factors for LNM and develop a nomogram which contained preoperative factors, including gastroscopy features, pathologic characteristics, tumor biomarkers, and radiology findings for individualized preoperative prediction of LNM in EGC patients.

## Materials and methods

### Patients

Data of this retrospective study was collected from Shanghai Changhai Hospital, China, which is a tertiary teaching hospital with approximately 2600 beds serving 140,000 inpatients and 2,200,000 outpatients and emergencies each year. Changhai Hospital is one of the largest national gastric carcinoma research centers in China with over 2000 GC patients treated per year.

**Fig. 1:**
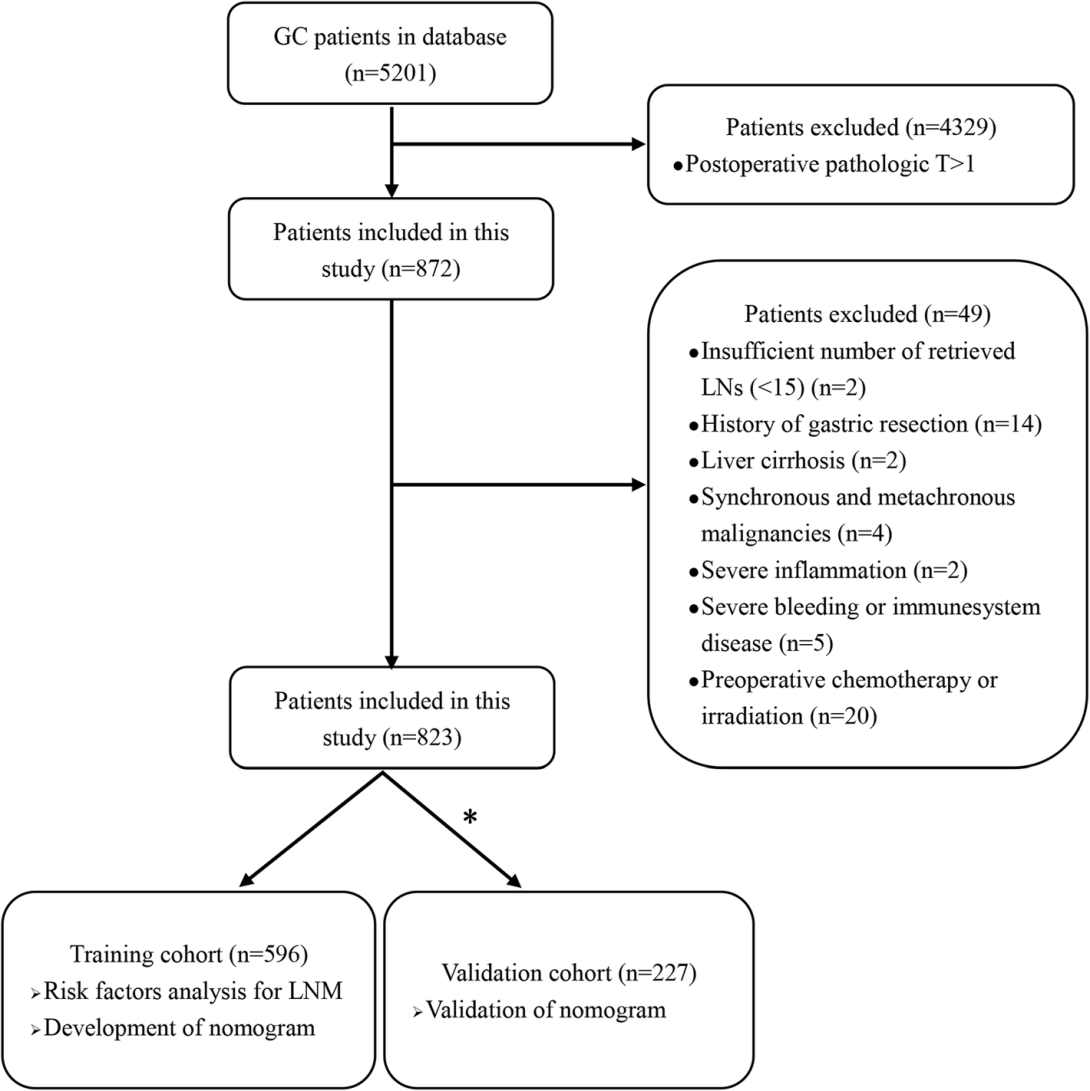
Flow diagram of patients enrollment and study design. *Patients admitted from 1 January 2015 to 31 December 2017 were included into the training cohort and from 1 January 2018 to 31 January 2019 into the validation cohort. GC: gastric cancer, LN: lymph node, LNM: lymph node metastasis

From 1 January 2015 to 1 January 2019, a total of 5201 GC patients were performed with radical gastrectomy and lymphadenectomy in our hospital, among which 872 patients with pT1a or pT1b in postoperative pathology were retrospectively analyzed in our study (Fig. 1). In total, 823 EGC patients were enrolled in the study, which included 596 EGC patients from 1 January 2015 to 31 December 2017 as the training cohort, and 227 patients from 1 January 2018 to 31 January 2019 as the independent validation cohort. Inclusion criteria were as follows: (a) patients who underwent surgery for GC with curative intent, (b) lymph nodes dissection performed, (c) preoperative gastroscopy findings available, (d) preoperative biopsy-proven histology differentiation and immunohistochemistry available, (e) plasma tumor biomarkers were tested within 10 days before surgical resection, and (f) standard contrast-enhanced computed tomography (CT) performed fewer than 10 days before surgical resection. Patients who had any one of the following features were excluded: (a) insufficient number of retrieved lymph nodes (< 15), (b) history of gastrectomy, (c) comorbid with cirrhosis, (d) synchronous and metachronous malignancies, (e) comorbid with severe inflammation, (f) comorbid with severe bleeding or diseases of the immune system, and (g) history of preoperative chemotherapy or irradiation. The number of patients excluded by each category was 2, 14, 2, 4, 2, 5, and 20 respectively.

### Clinicopathologic characteristics

Preoperative gastroscopy was performed with the goal of determining the location, size, and macroscopy features of the tumor, and the results were recorded in standardized tables. The location of tumor was categorized as cardia, corpus/fundus, and antrum/angularis/pylorus. Tumor size which means the maximum diameter of tumor was recorded with continuous variable, then converted to a classification variable with the threshold value < 2.20 cm and ≥ 2.20 cm, analyzed by optimal binning, which was consistent with previous studies [14, 16]. The macroscopic feature was classified into three groups: elevated type (types I and IIa), flat type (type IIb), or depressed type (types IIc and III). Multiple biopsies were performed to provide adequate material for histologic interpretation. Histology differentiation was classified as well, moderated or poorly. Immunohistochemistry of the biopsy included Topo II, P53, and Ki67. The percentage of positive tumor cells > 10% were defined as positivity [16–18]. Routine preoperative laboratory measurements of tumor markers including carcinoembryonic antigen (CEA), carbohydrate antigen 72-4 (CA72-4), and carbohydrate antigen 19-9 (CA19-9) were recorded in our study, among which CA19-9 was converted to a classification variable with the threshold value < 36.78 U/mL and ≥ 36.78 U/mL, analyzed by optimal binning, which was exactly the normal value (37 U/mL) in our center. CEA and CA72-4 were unable to create bins because of weak or no association and then were converted to a classification variable by a normal range. Standard contrast-enhanced CT was performed less than 10 days before surgical resection, and predictions of the presence of LNM by CT scans were recorded.

### Statistical analysis

All of the data were analyzed using the SPSS 23.0 statistical package (SPSS Inc., Chicago, IL, USA) and R software (version 3.5.2; http://www.Rproject.org). The significance level for all of the statistical tests was set at 0.05. All statistical tests were two-sided.

Continuous values were analyzed with mean and standard deviation. Student’s *t* test was applied to compare continuous variables, and chi-square test (or Fisher’s exact test in specific condition) was applied to analyze categorical variables when comparing differences among various groups. Binary logistic regression modeling technique was applied to analyze risk factors for LNM. All variables that had a *p* value of < 0.05 in univariate analysis were selected into the multivariable logistic analysis to further identify independent risk factors. In multivariable logistic analysis, variables with a *p* value of < 0.05 were identified to be independent risk factors and selected into the final model and those without statistical significance were excluded from the final model automatically.

### Development and validation of the nomogram

To develop a quantitative and relatively accurate tool to predict the individual probability of LNM, a nomogram was developed on the basis of all independent risk factors identified by multivariable logistic analysis in the training cohort via using the rms package of R software. Only independent risk factors identified by multivariable logistic analysis were selected into the final model to build the nomogram and variables which were statistically significant in univariate analysis but were not statistically significant in multivariable analysis were not selected during the nomogram development. In the nomogram, the regression coefficient of each independent risk factor in multivariate logistic regression was proportionally converted to a specific number within a 0- to 100-point scale. To evaluate the internal and external discrimination performance of the nomogram, bootstrapping validation (1000 bootstrap resamples) was carried out based on the training and validation cohort, separately. The discrimination which represented the predictive accuracy of the nomograms was evaluated by concordance index (C index) and the calibration curves for both internal and external validation.

## Results

### Clinicopathological features of patients

The clinicopathological features of EGC patients in both the training and validation cohorts are exhibited in Table 1. LNM rate in the training cohort was 16.4%, and it was 15.4% in validation cohort (*P* = 0.752). No significant differences was observed in terms of the basic clinical characteristics between training and validation cohort, either within the lymph node-positive or the lymph node-negative group, which verified the training and validation cohorts had homogeneous baseline data.

**Table 1:** Characteristics of patients in the training and validation cohorts

Variables	Training cohort			Validation cohort		
	Overall (*n* = 596)	LNM(+) (*n* = 98)	LNM(−) (*n* = 498)	Overall (*n* = 227)	LNM(+) (*n* = 35)	LNM(−) (*n* = 192)
Age, no. (%)						
< 60 years	271 (45.5)	44 (44.9)	227 (45.6)	104 (45.8)	16 (15.7)	88 (45.8)
≥ 60 years	325 (54.5)	54 (55.1)	271 (54.4)	123 (54.2)	19 (54.3)	104 (54.2)
BMI, no. (%)						
< 18.5 kg/m^2^	39 (6.5)	12 (12.2)	27 (5.4)	18 (7.9)	1 (2.9)	17 (8.9)
≥ 18.5 kg/m^2^, < 25 kg/m^2^	373 (62.6)	57 (58.2)	316 (63.5)	145 (63.9)	26 (74.3)	119 (62.0)
≥ 25 kg/m^2^	184 (30.9)	29 (29.6)	155 (31.1)	64 (28.2)	8 (22.9)	54 (29.2)
Gender, no. (%)						
Female	179 (30.0)	30 (30.6)	149 (29.9)	72 (31.7)	8 (22.9)	64 (33.3)
Male	417 (70.0)	68 (69.4)	349 (70.1)	115 (68.3)	27 (77.1)	128 (66.7)
Tumor size level, no. (%)						
< 2.20 cm	390 (65.4)	42 (42.9)	348 (69.9)	154 (67.8)	20 (57.1)	134 (69.8)
≥ 2.20 cm	206 (34.6)	56 (57.1)	150 (30.1)	73 (32.2)	15 (42.9)	58 (30.2)
Tumor location, no. (%)						
Cardia	69 (11.6)	4 (4.1)	65 (13.1)	26 (11.5)	0 (0)	26 (13.5)
Corpus/fundus	121 (20.3)	24 (24.5)	97 (19.5)	48 (21.1)	9 (25.7)	39 (20.3)
Antrum/angularis/pylorus	406 (68.1)	70 (71.4)	336 (67.4)	153 (67.4)	26 (74.3)	127 (66.1)
Macroscopic appearance, no. (%)						
Elevated type (I + IIa)	113 (19.0)	18 (18.4)	95 (19.1)	33 (14.5)	3 (8.6)	30 (15.6)
Flat type (IIb)	201 (33.7)	20 (20.4)	181 (36.3)	73 (32.2)	7 (20.0)	66 (34.4)
Depressed type (IIc + III)	282 (47.3)	60 (61.2)	222 (44.6)	121 (53.3)	25 (71.4)	96 (50.0)
Histology differentiation, no. (%)						
Well	58 (9.7)	1 (1.0)	57 (11.4)	19 (8.4)	1 (2.9)	18 (9.4)
Moderate	319 (53.5)	42 (42.9)	277 (55.6)	127 (55.9)	16 (45.7)	111 (57.8)
Poorly	219 (36.7)	55 (56.1)	164 (32.9)	81 (35.7)	18 (51.4)	63 (32.8)
Topo II, no. (%)						
Negative	248 (41.6)	46 (49.6)	202 (40.6)	88 (38.8)	18 (51.4)	70 (36.5)
Positive	348 (58.4)	52 (53.1)	296 (59.4)	139 (61.2)	17 (48.6)	122 (63.5)
P53, no. (%)						
Negative	353 (59.2)	47 (48.0)	306 (61.4)	138 (60.8)	14 (40.0)	124 (64.6)
Positive	342 (40.8)	51 (52.0)	192 (38.6)	89 (39.2)	21 (60.0)	68 (35.4)
Ki67, no. (%)						
Negative	18 (3.0)	1 (1.0)	17 (3.4)	9 (4.0)	1 (2.9)	8 (4.2)
Positive	578 (97.0)	97 (99.0)	481 (96.6)	218 (96.0)	34 (97.1)	184 (95.8)
CEA, no. (%)						
<5 ng/mL	551 (92.4)	86 (87.8)	465 (93.4)	210 (92.5)	30 (85.7)	180 (93.8)
≥ 5 ng/mL	45 (7.6)	12 (12.2)	33 (6.6)	17 (7.5)	5 (14.3)	12 (6.3)
CA19-9 level, no. (%)						
<36.78 U/mL	570 (95.6)	81 (82.7)	489 (98.2)	219 (96.5)	29 (82.9)	190 (99.0)
≥ 36.78 U/mL	26 (4.4)	17 (17.3)	9 (1.8)	8 (3.5)	6 (17.1)	2 (1.0)
CA72-4, no. (%)						
<9.8 U/mL	502 (84.2)	81 (82.7)	421 (84.5)	199 (87.7)	29 (82.9)	170 (88.5)
≥ 9.8 U/mL	94 (15.8)	17 (17.3)	77 (15.5)	28 (12.3)	6 (17.1)	22 (11.5)
CT-reported LN status, no. (%)						
Negative	475 (79.7)	60 (61.2)	415 (83.3)	184 (81.1)	25 (71.4)	159 (82.8)
Positive	121 (20.3)	38 (38.8)	83 (16.7)	43 (18.9)	10 (28.6)	33 (17.2)
LN retrieved, mean ± SD, no.	24.980 ± 7.803	26.163 ± 8.891	24.747 ± 7.559	25.282 ± 8.384	26.571 ± 9.172	25.047 ± 8.236

*BMI* body mass index, *CA19-9* carbohydrate antigen 19-9, *CA72-4* carbohydrate antigen 72-4, *CEA* carcinoembryonic antigen, *CT* computed tomography, *LN* lymph node, *LNM* lymph node metastasis, *SD* standard deviation

In training cohort, the tumor size was 2.048 ± 1.253 cm, 34.6% in larger size tumors (≥ 2.20 cm). In total, 11.6%, 20.3%, and 68.1% of the tumors were located in the cardia, corpus/fundus, and antrum/angularis/pylorus respectively. In the macroscopic appearance, 19.0% was elevated type, 33.7% was flat type, and 47.3% was depressed type. In histology differentiation, the ratios of well, moderate, and poorly grade were 9.7%, 53.5% and 36.7%, respectively. The ratios of Topo II (+), P53 (+), and Ki67 (+) were 41.6%, 59.2%, and 3.0%, respectively. The ratios of CEA, CA19-9, and CA72-4 above the normal range were 7.6%, 4.4%, and 15.8%, respectively. CT-reported lymph node positive status was 20.3%.

### Predictors for LNM in EGC patients

The univariate and multivariable logistic regression analyses are summarized in Table 2. In univariate analysis, seven variables, which included tumor size, tumor location, macroscopic appearance, histology differentiation, P53, CA19-9, and CT-reported lymph node status showed *P* values of less than 0.05.

**Table 2:** Predictive factors for LNM in EGC patients (596 cases)

Predictors	*n* (%)	Univariate analysis		Multivariable analysis^*^	
		*P*	OR (95% CI)	*P*	OR (95% CI)
Age, no. (%)		0.901			
< 60 years	271 (45.5)		Reference		
≥ 60 years	325 (54.5)		1.03 (0.67–1.59)		
BMI, no. (%)		0.052			
< 18.5 kg/m^2^	39 (6.5)		2.38 (1.08–5.22)		
≥ 18.5 kg/m^2^, < 25 kg/m^2^	373 (62.6)		0.96 (0.59–1.57)		
≥ 25 kg/m^2^	184 (30.9)		Reference		
Gender, no. (%)		0.891			
Female	179 (30.0)		Reference		
Male	417 (70.0)		0.97 (0.61–1.55)		
Tumor size level, no. (%)		< 0.001		< 0.001	
< 2.20 cm	390 (65.4)		Reference		Reference
≥ 2.20 cm	206 (34.6)		3.09 (1.99–4.82)		3.18 (1.91–5.30)
Tumor location, no. (%)		0.046			
Cardia	69 (11.6)		Reference		
Corpus/fundus	121 (20.3)		4.02 (1.33–12.13)		
Antrum/angularis/pylorus	406 (68.1)		3.39 (1.19–9.60)		
Macroscopic feature, no. (%)		0.005		0.027	
Flat type (IIb)	201 (33.7)		Reference		Reference
Elevated type (I + IIa)	113 (19.0)		1.72 (0.87–3.40)		1.80 (0.85–3.80)
Depressed type (IIc + III)	282 (47.3)		2.45 (1.42–4.21)		2.29 (1.25–4.20)
Histology grades, no. (%)		< 0.001		< 0.001	
Well	58 (9.7)		Reference		Reference
Moderate	319 (53.5)		8.64 (1.17–64.09)		8.75 (1.11–68.78)
Poorly	219 (36.7)		19.12 (2.59–141.32)		30.76 (3.85–245.97)
Topo II, no. (%)		0.243			
Negative	248 (41.6)		Reference		
Positive	348 (58.4)		0.77 (0.50–1.19)		
P53, no. (%)		0.014		< 0.001	
Negative	353 (59.2)		Reference		Reference
Positive	342 (40.8)		1.73 (1.12–2.67)		3.32 (1.93–5.72)
Ki67, no. (%)		0.234			
Negative	18 (3.0)		Reference		
Positive	578 (97.0)		3.43 (0.45–26.07)		
CEA, no. (%)		0.058			
< 5 ng/mL	551 (92.4)		Reference		
≥ 5 ng/mL	45 (7.6)		1.97 (0.98–3.96)		
CA19-9 level, no. (%)		< 0.001		< 0.001	
< 36.78 U/mL	570 (95.6)		Reference		Reference
≥ 36.78 U/mL	26 (4.4)		11.40 (4.92–26.45)		9.63 (3.75–24.72)
CA72-4, no. (%)		0.640			
< 9.8 U/mL	502 (84.2)				
≥ 9.8 U/mL	94 (15.8)		1.15 (0.65–2.04)		
CT-reported LN status, no. (%)		< 0.001		< 0.001	
Negative	475 (79.7)		Reference		Reference
Positive	121 (20.3)		3.17 (1.98–5.07)		2.91 (1.69–5.00)

^*^In multivariable analysis, tumor size, tumor location, macroscopic appearance, histology differentiation, P53, CA19-9, and CT-reported lymph node status were adjusted in the multivariable analyses

*BMI* body mass index, *CA19-9* carbohydrate antigen 19-9, *CA72-4* carbohydrate antigen 72-4, *CEA* carcinoembryonic antigen, *CT* computed tomography, *LN* lymph node, *LNM* lymph node metastasis

In multivariable analysis, seven risk factors aforementioned were included in the logistic regression model. Finally, the larger tumor size (OR 3.22, 95% CI, 1.93–5.35), P53 (OR 5.46, 95% CI, 2.47–12.07), higher CA19-9 level (OR 9.25, 95%CI, 3.66–23.34), and CT-reported LN status (OR 2.79, 95%CI, 1.63–4.78) were proved to be independent risk factors for LNM. And moderate differentiation (OR 7.43, 95%CI, 0.95–58.06) and poor differentiation (OR 26.02, 95%CI, 3.30–205.41) were identified as risk factors compared to well differentiation. Meanwhile, flat type (OR 0.55, 95%CI, 0.27–1.23) and depressed type (OR = 1.27, 95%CI, 0.66–2.44) were protective and risk factors respectively compared to elevated type.

### Development and validation of the nomogram for the prediction of LNM

**Fig. 2:**
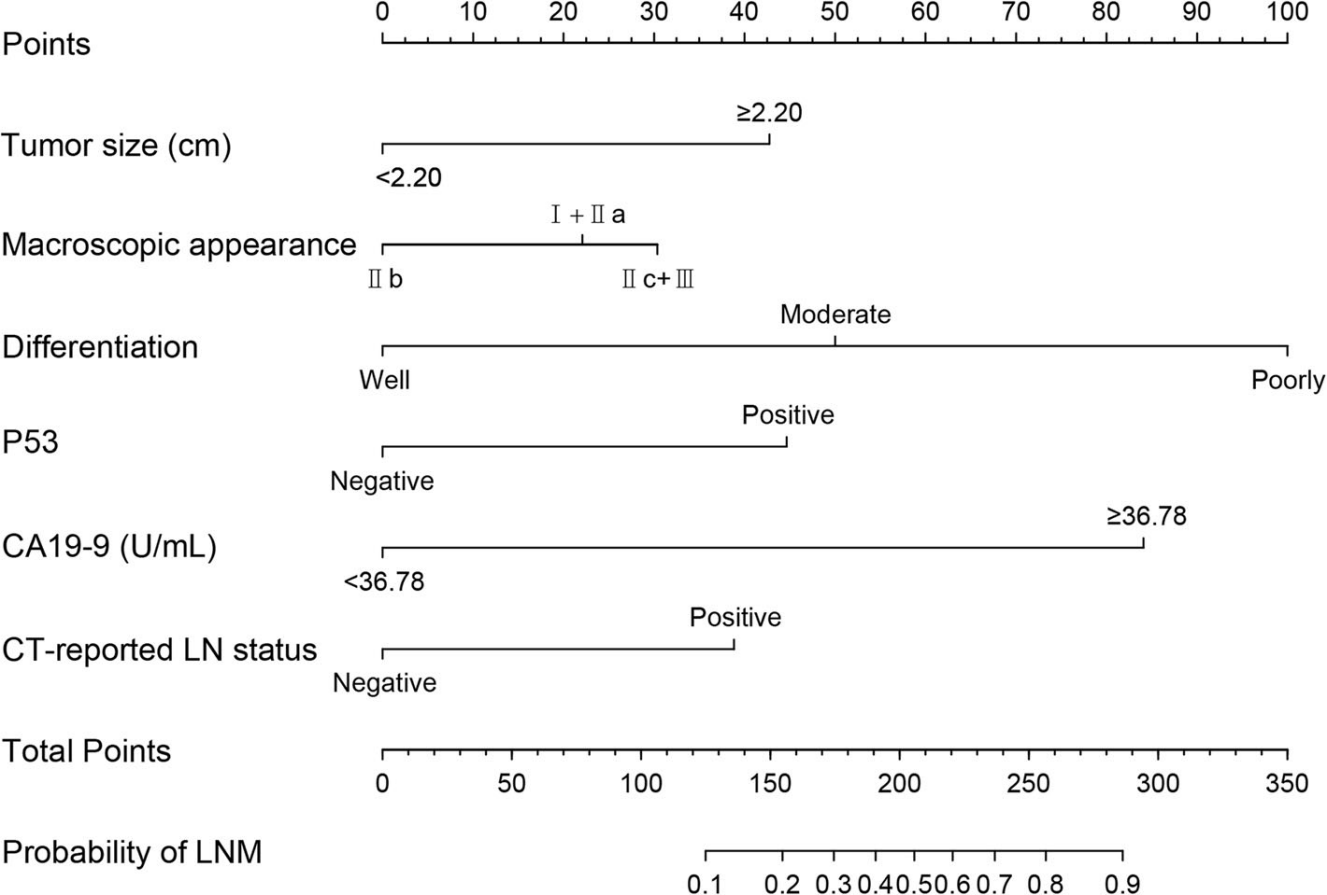
Nomogram for preoperative prediction of lymph node metastasis in early gastric cancer. The probability of lymph node metastasis involvement in early gastric cancer is calculated by (1) drawing a line to an axis on each of the following variables: tumor size, macroscopic appearance, histologic differentiation, P53, CA19-9, and CT-reported lymph node status, (2) adding the points of each variable and locate them on the total point line, then (3) obtaining the individual probability of lymph node metastasis by projecting the vertical line from the total point line to the bottom scale of the prediction probability. CA19-9: carbohydrate antigen 19-9, CT: computed tomography, LN: lymph node, LNM: lymph node metastasis

Six independent risk factors were enrolled to develop LNM prediction nomogram (Fig. 2). When applying the nomogram, the point for each predictor was analyzed through drawing a straight line upward from each predictor with the specific status to the “Point” axis. The total points for an individual patient were calculated by summing up all the separate points for the six predictors. The estimated incidence of LNM in EGC patients can be finally determined by drawing a straight line down from the “Total Point” axis to the “Probability of LNM” axis.

**Fig. 3:**
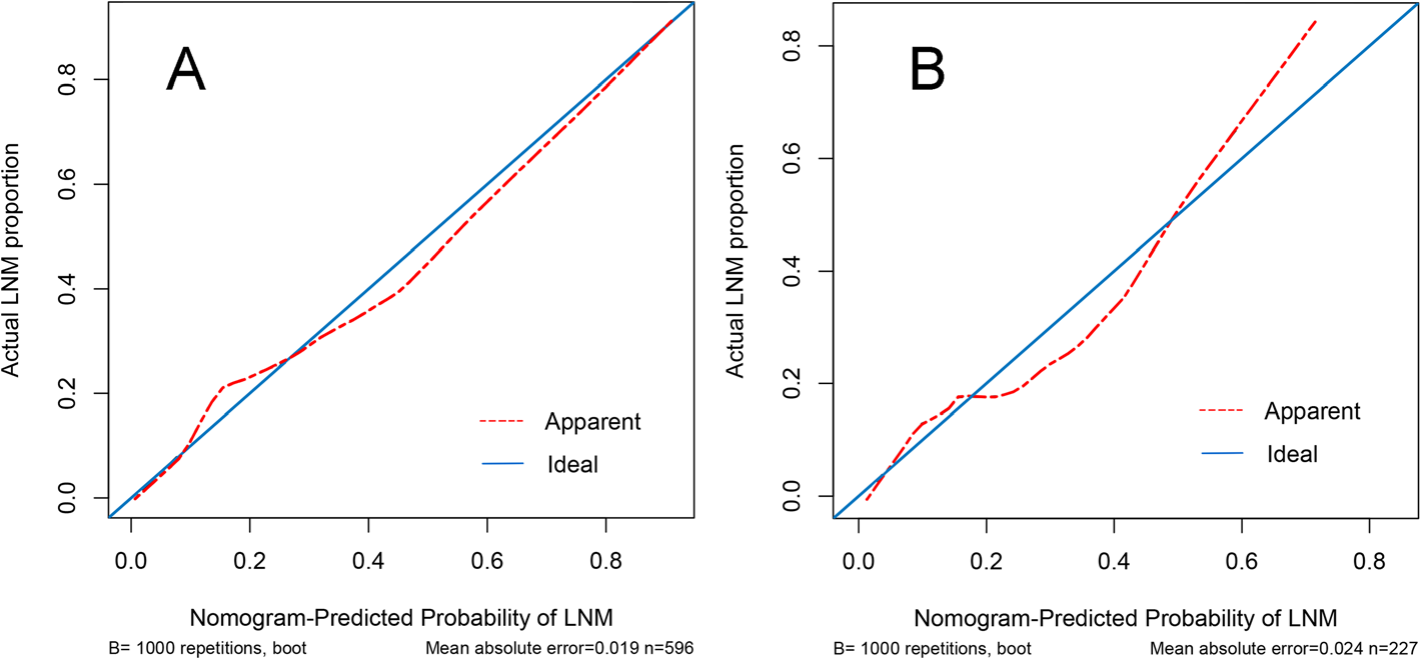
**a**, **b** Validity of the predictive performance of the nomogram in estimating the risk of lymph node metastasis in early gastric cancer patients. **a** Internal calibration curve to validate the nomogram model and the C-index was 0.82 (95%CI, 0.78 to 0.80). **b** External calibration curve to validate the nomogram model and the C-index was 0.77 (95%CI, 0.60 to 0.94)

Furthermore, an internal calibration curve was developed (Fig. 3a) to validate the nomogram model and the C-index was 0.82 (95%CI, 0.78 to 0.86), which showed a good discrimination and calibration. The predictive accuracy of the nomogram was then evaluated by the validation cohort (Fig. 3b). In this external validation, the C-index was 0.77 (95%CI, 0.60 to 0.94), implying a good concordance.

## Discussion

This is the first and the only study which developed a nomogram to predict the probability of LNM in EGC patients according to preoperative factors as far as we know. Tumor size, macroscopic appearance, histology differentiation, P53, CA19-9, and CT could all be easily obtained from preoperative routine examinations. Tumor size and macroscopic appearance were observed and measured by gastroscopy. Histology differentiation could be obtained by pathological examination of biopsy, and P53 was obtained by immunohistochemistry. CA19-9 was the regular tumor marker from plasma. CT was also the regular examination in EGC patients. All these factors were easy and convenient to obtain in EGC patients, so this model had good application in clinical practice. This nomogram could predict the incidence of LNM for every individual patient, which could help both clinicians and patients to make a wise and customized decision in clinical treatment. For the development of nomogram, firstly we analyzed the clinical characteristics of the training cohort. This nomogram was verified to have good discrimination both in the training cohort (C-index, 0.82) and validation cohort (C-index, 0.77).

Lager tumor size, depressed type, and poor differentiation were proved to be independent risk factors in the present study which coincides with previous studies [19–21]. Lager tumor size, depressed type, and poor differentiation indicated worse biological behavior, which might also indicate higher probability of LNM.

Three immunohistochemical markers were analyzed to explore the relationship between LNM and tumor markers in EGC. However, only P53, an important tumor suppressor gene, turned out to be an independent risk factor of LNM in EGC. The mutation of P53 results in the change of its spatial conformation, and the loss of the function of regulation of cell growth, apoptosis and DNA repair [16]. Several studies had already proved that P53 was related to carcinogenesis and poor prognosis in patients with GC [22, 23]. It was reported that Topo II and Ki67 could reflect the proliferation activity of cancer cells and affect the postoperative recurrence in breast cancer [24]. However, several studies had proved that Topo II was not associated with LNM [16, 25]. And the value of Ki67 in predicting LNM of EGC varies differently in some articles [26, 27]. In our study, the results revealed that Topo II and Ki67 were insignificantly associated with LNM in EGC.

Tumor biomarkers, which can reflect the occurrence and development of tumors, can also be easily obtained. A previous study had demonstrated that CEA and CA19-9 were independent predictive factors of liver metastasis of colorectal cancer through LNM [28]. Also, several studies had illustrated there was association between elevation of the CA19-9 and CA72-4 levels and presence of LNM in EGC patients, and elevation of the CEA level was proved to be an independent predictor for the poor prognosis of EGC [29, 30]. However, a study indicated that CEA was unrelated to LNM in EGC [31]. In this study, only CA19-9 was associated with LNM in EGC.

CT scan is commonly used to evaluate the lymph nodes status, which appears to be one of the most reliable tools in clinical practice. Previous studies reported the accuracy rate of CT evaluation was around 60% [28, 32]. Recent studies suggested that magnetic resonance imaging (MRI) might be beneficial for treatment response assessment and systemic diseases; however, it was no better in the diagnosis of regional LNM than CT [33, 34]. The sensitivity of positron emission tomography-computed tomography (PET-CT) in evaluating regional LNM in EGC still remained controversial [35, 36]. However, since the cost of PET-CT was expensive, it was not a regular examination for patients. Thus, CT is still the most reliable tool in current practice [19]. In our study, CT was proved to be an independent predictor of LNM in EGC.

In previous studies, several nomograms had been built for this purpose, but some factors they used were not preoperatively available information which could only be obtained postoperatively. This might have limited the application of those nomograms from previous studies in clinical practice. In our study, we obtained six preoperative factors from regular examinations and developed a new kind of nomogram to predict LNM in EGC patients. This nomogram model was convenient to apply, and it was also proved to have a high discrimination for predicting LNM in EGC patients.

Several limitations exist in the present study. Firstly, this is a retrospective study in which the patients were from a single center. Though we had 596 patients in the training cohort and 227 patients in the validation cohort, more data are needed, especially from other centers, to evaluate the applicability of the results from this study in an external population. Secondly, because of the histological heterogeneity which was one of the distinctive features of GC, discrepancy often exists between preoperative and postoperative histology results. Usually, the amount of tissues, mainly from the mucosa, obtained by biopsy is limited. However, the reported percentage of histological differences in EGC was between 9.4 and 16.3% [37–39], which was acceptable. Thirdly, it was not easy to develop a cutoff to stratify patients with a high rate LNM. A false negative outcome was much more dangerous than a false positive outcome. Underestimating the risk of tumor led to a more serious outcome than overestimating the risk of tumor. So before making treatment decision, we recommend a careful discussion with patients. The cutoff point depended on how the patients and doctors reject risk. Therefore, this nomogram is more useful to provide patients and doctors with evidence than stratification. Finally, there was a selection bias because EGC patients who underwent ESD included those required subsequent surgery only.

## Conclusion

Our study presents a new nomogram which incorporated only preoperative factors, which could be used to identify EGC patients with the high risk of LNM, hence helping clinicians and patients make a wise choice before operation.

## Data Availability

The datasets used and/or analyzed during the current study are available from the corresponding author on reasonable request.

## Abbreviations

CA19-9: Carbohydrate antigen 19-9
CA72-4: Carbohydrate antigen 72-4
CEA: Carcinoembryonic antigen
CT: Computed tomography
EGC: Early gastric cancer
EMR: Endoscopic mucosal resection
ESD: Endoscopic submucosal dissection
GC: Gastric cancer
LNM: Lymph node metastasis
MRI: Resonance imaging
OR: Odds ratio
PET-CT: Positron emission tomography-computed tomography
